# Supplementary material for: Asymmetric responses to simulated global warming by populations of Colobanthus quitensis along a latitudinal gradient
Source: PeerJ. 2017 Sep 18;5:e3718. doi: 10.7717/peerj.3718 (PMC5607920; doi:10.7717/peerj.3718)
Supplement: Table S1 — The net photosynthetic performance (measured as net photosynthesis rate) is shown under each thermal condition and population in common gardens and the ecophysiological responses to the simulated global warming expressed as the delta values between future and current individual values. [file peerj-05-3718-s001.docx]

**Supplementary material**

**Table S1**: Summary of the results of independent one-way ANOVAs evaluating the effect of latitudinal origin (either population or temperature) on anatomical foliar traits and xanthophyll cycle pigment content of *C. quitensis* individuals sampled in the field. The net photosynthetic performance (measured as net photosynthesis rate) is shown under each thermal condition and population in common gardens and the ecophysiological responses to the simulated global warming expressed as the delta values between future and current individual values.

| **Trait** | **Factor** | ***df*** | ***SS*** | ***MS*** | ***F*** | ***P*** |
| --- | --- | --- | --- | --- | --- | --- |
| ***Microstructures*** | | | | | | |
| Cuticle width (µm) | Pop | 2,72 | 829.80 | 414.90 | 107.70 | **< 0.001** |
| Mesophill width (µm) | Pop | 2,72 | 114662 | 57331 | 225.90 | **< 0.001** |
| Palisade Parenchyma width (µm) | Pop | 2,72 | 3367 | 1683.50 | 165.40 | **< 0.001** |
| Spongy Mesophyll width (µm) | Pop | 2,72 | 97392 | 48696 | 174.60 | **< 0.001** |
| Leaf transversal area (mm^2^) | Pop | 2,72 | 0.45 | 0.23 | 513.20 | **< 0.001** |
| ***Pigments*** |  |  |  |  |  |  |
| Anteraxanthin (µg g^-1^ DW) | Pop | 2,42 | 217.80 | 108.90 | 5.19 | **0.001** |
| Violaxanthin (µg g^-1^ DW) | Pop | 2,42 | 50.71 | 25.35 | 5.88 | **0.005** |
| Zeaxanthin (µg g^-1^ DW) | Pop | 2,42 | 3154 | 1577 | 52.69 | **< 0.001** |
| DEPS (µg g^-1^ DW) | Pop | 2,42 | 0.04 | 0.02 | 36.83 | **< 0.001** |
| ***Experimental warming*** | | | | | | |
| Net photosynthesis (µm CO_2_ m^-2^s^-1^) | Pop | 2,42 | 47.26 | 23.63 | 33.78 | **< 0.001** |
| Biomass accumulation (g) | Pop | 2,42 | 16.31 | 8.15 | 4.29 | **0.020** |
| Flower production (n) | Pop | 2,42 | 9.64 | 4.82 | 3.79 | **0.031** |
