# Supplementary material for: Asymmetric responses to simulated global warming by populations of Colobanthus quitensis along a latitudinal gradient
Source: PeerJ. 2017 Sep 18;5:e3718. doi: 10.7717/peerj.3718 (PMC5607920; doi:10.7717/peerj.3718)
Supplement: Table S2 — Bonferroni correction was applied to all P values due to multiple comparisons. [file peerj-05-3718-s002.docx]

**Supplementary material**

**Table S2:** Pair-wise *a posteriori* comparisons (HSD Tukey test’s) between the net photosynthetic response of each population under current (t0) and future conditions estimated during three simulated growing seasons (t_1_:t_3_). Bonferroni correction was applied to all *P* values due to multiple comparisons.

| **South America** | |  |  |  |
| --- | --- | --- | --- | --- |
|  | **Estimate** | **Std. Error** | **z value** | **P-value** |
| t1-t0 | 0.70 | 0.13 | 5.57 | **<0.001** |
| t2-t0 | 0.89 | 0.13 | 7.10 | **<0.001** |
| t3-t0 | 0.85 | 0.13 | 6.76 | **<0.001** |
| t2-t1 | 0.19 | 0.13 | 1.53 | 0.422 |
| t3-t1 | 0.15 | 0.13 | 1.19 | 0.631 |
| t3-t2 | -0.04 | 0.13 | -0.33 | 0.987 |
|  |  |  |  |  |
| **Shetland Islands** | |  |  |  |
|  | **Estimate** | **Std. Error** | **z value** | **P-value** |
| t1-t0 | 0.83 | 0.11 | 7.57 | **<0.001** |
| t2-t0 | 1.13 | 0.11 | 10.24 | **<0.001** |
| t3-t0 | 1.25 | 0.11 | 11.47 | **<0.001** |
| t2-t1 | 0.29 | 0.11 | 2.68 | **0.038** |
| t3-t1 | 0.43 | 0.11 | 3.89 | **<0.001** |
| t3-t2 | 0.13 | 0.11 | 1.22 | 0.612 |
|  |  |  |  |  |
| **Anarctic Peninsula** | |  |  |  |
|  | **Estimate** | **Std. Error** | **z value** | **P-value** |
| t1-t0 | 2.31 | 0.16 | 14.83 | **<0.001** |
| t2-t0 | 3.19 | 0.16 | 20.51 | **<0.001** |
| t3-t0 | 3.33 | 0.16 | 21.37 | **<0.001** |
| t2-t1 | 0.88 | 0.16 | 5.68 | **<0.001** |
| t3-t1 | 1.02 | 0.16 | 6.53 | **<0.001** |
| t3-t2 | 0.13 | 0.16 | 0.86 | 0.827 |
