# Supplementary material for: Asymmetric responses to simulated global warming by populations of Colobanthus quitensis along a latitudinal gradient
Source: PeerJ. 2017 Sep 18;5:e3718. doi: 10.7717/peerj.3718 (PMC5607920; doi:10.7717/peerj.3718)
Supplement: Table S3 — Mean, standard deviation (SD) and standard error (SE) are shown. [file peerj-05-3718-s003.docx]

**Supplementary material**

**Table S3:** Descriptive statistic of net photosynthesis measured (μmol CO2 m^-2^s^-1^) of each population under current (t_0_) and future conditions estimated during three simulated growing seasons (t_1_:t_3_). Mean, standard deviation (SD) and standard error (SE) are shown.

| **Population** | **Statistic** | **Current**  **t_0_** | **Future**  **t_1_** | **Future**  **t_2_** | **Future**  **t_3_** |
| --- | --- | --- | --- | --- | --- |
| South America | **Mean** | **7.03** | **7.92** | **8.11** | **8.07** |
|  | SD | 0.38 | 0.27 | 0.38 | 0.23 |
|  | SE | 0.10 | 0.07 | 0.10 | 0.06 |
| Shetland Island | **Mean** | **4.19** | **5.08** | **5.38** | **5.51** |
|  | SD | 0.43 | 0.28 | 0.12 | 0.12 |
|  | SE | 0,11 | 0.07 | 0.03 | 0.03 |
| Antarctic Peninsula | **Mean** | **3.90** | **6.11** | **6.99** | **7.12** |
|  | SD | 0.34 | 0.37 | 0.54 | 0.31 |
|  | SE | 0.09 | 0.10 | 0.14 | 0.08 |
